# The final stages of the global eradication of poliomyelitis

**DOI:** 10.1098/rstb.2012.0140

**Published:** 2013-08-05

**Authors:** Nicholas C. Grassly

**Affiliations:** Department of Infectious Disease Epidemiology, Imperial College London, Norfolk Place, London W2 1PG, UK

**Keywords:** poliovirus, poliomyelitis, polio, eradication, vaccine, public health

## Abstract

The global incidence of poliomyelitis has dropped by more than 99 per cent since the governments of the world committed to eradication in 1988. One of the three serotypes of wild poliovirus has been eradicated and the remaining two serotypes are limited to just a small number of endemic regions. However, the Global Polio Eradication Initiative (GPEI) has faced a number of challenges in eradicating the last 1 per cent of wild-virus transmission. The polio endgame has also been complicated by the recognition that vaccination with the oral poliovirus vaccine (OPV) must eventually cease because of the risk of outbreaks of vaccine-derived polioviruses. I describe the major challenges to wild poliovirus eradication, focusing on the poor immunogenicity of OPV in lower-income countries, the inherent limitations to the sensitivity and specificity of surveillance, the international spread of poliovirus and resulting outbreaks, and the potential significance of waning intestinal immunity induced by OPV. I then focus on the challenges to eradicating all polioviruses, the problem of vaccine-derived polioviruses and the risk of wild-type or vaccine-derived poliovirus re-emergence after the cessation of oral vaccination. I document the role of research in the GPEI's response to these challenges and ultimately the feasibility of achieving a world without poliomyelitis.

## 1. Introduction

Poliovirus is a small RNA virus, just 30 nm across and with a complete genome of only approximately 7500 nucleotides. It is shed in enormous quantities in the throat and intestines of infected individuals such that a gram of stool can contain several million virus particles [1]. In settings with faecal contamination of the environment and water supplies the estimated basic reproduction number is very high (perhaps 10–15; [2]). The global eradication of wild-type poliovirus therefore represents a major technical and political challenge. Yet the world committed to eradication at the World Health Assembly in 1988 [3]. At that time polio was endemic in 125 countries and it has been estimated by the WHO that more than 350 000 children each year developed poliomyelitis [4].

Combined with improved surveillance of children with acute flaccid paralysis, mass vaccination with oral poliovirus vaccine (OPV) in areas with weak health systems has allowed the Global Polio Eradication Initiative (GPEI) to eliminate wild-type polioviruses from much of the world. The GPEI has exclusively relied on OPV, rather than the injected inactivated poliovirus vaccine (IPV), because of its ease of administration and superior ability to induce intestinal mucosal immunity against infection and transmission of poliovirus in stool [5]

**Figure 1.**
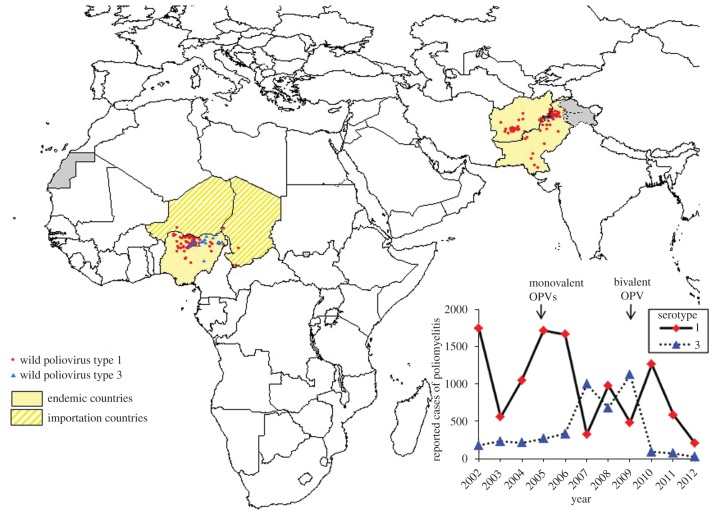
The geographical distribution of children with poliomyelitis associated with wild-type poliovirus shown by serotype for 2012 and (inset) the total number of children with poliomyelitis globally by serotype reported each year during 2001–2012. Poliomyelitis as a result of vaccine-derived polioviruses is not shown. India was declared ‘polio-free’ in 2012, the last case reported from West Bengal in January 2011. The arrows above the inset graph indicate when monovalent and bivalent OPVs were first used by the GPEI. Map and data are from WHO (www.polioeradication.org).

The original target date for the global eradication of poliomyelitis was the year 2000. The GPEI came close, with only six countries remaining endemic for polio and just under 3000 children paralysed by polio that year. Perhaps even more encouraging was the successful eradication of wild-type 2 poliovirus—the last naturally occurring case was reported from India in 1999—leaving two serotypes still in circulation [1,3]. Over the next decade polio was eliminated from Egypt and Niger, but persisted in four countries—Pakistan, Afghanistan, India and Nigeria—despite extensive efforts by the GPEI. Wild poliovirus from these countries travelled to other countries, particularly in Africa, resulting in over 50 outbreaks and costing the programme several hundred million dollars in outbreak control activities. As a result, case numbers remained stubbornly at approximately one or two thousand each year. In 2012, however, the number of reported cases reached an all-time low of 223, following successful elimination from India, where the last case of poliomyelitis due to indigenous virus occurred in January 2011 [6] (figure 1).

Major challenges have emerged and many conquered during the course of the last decade. Most significantly, it was recognized that OPV can, in rare instances, evolve to regain wild-type transmissibility and pathogenicity, and can result in large outbreaks of vaccine-derived polioviruses [7,8]. This recognition made it clear that after the eradication of wild-type poliovirus, vaccination with OPV would have to be stopped in a coordinated fashion to prevent the creation of new vaccine-derived poliovirus outbreaks [9].

In this paper, I describe the challenges to polio eradication over the last decade or so, and how they have been approached and in most cases overcome. I then describe the challenges to be faced over the coming years, particularly post-eradication of wild-type polioviruses when OPV can no longer be used. A summary of these major challenges and outstanding research needs is provided in table 1.

**Table 1.** Major technical challenges faced by the Global Polio Eradication Initiative and outstanding research needs.

challenge	response	research needs
poor OPV immunogenicity	introduction of monovalent and bivalent OPVs	an understanding of why OPV is less immunogenic in lower-income countries
		further improvements in OPV immunogenicity
achieving high OPV coverage	improved post-campaign monitoring	innovations in vaccine delivery (e.g. GIS for campaign microplans)
	new management practices	improved campaign monitoring (e.g. mobile phone technology)
	political advocacy	
	community engagement	
	increased technical assistance	
	strengthened routine immunization	
	innovations in vaccine delivery (e.g. short-interval campaigns, fixed vaccination posts, etc.)	
surveillance sensitivity and timeliness	faster laboratory protocols	cheap methods to detect poliovirus in clinical and environmental samples without the need for cell culture (faster and safer)
	expanded environmental surveillance	
	improved sensitivity to detect vaccine-derived polioviruses (new primers)	improved tools to collect and process large numbers of environmental samples
emerging immunity gaps and polio outbreaks	risk assessment and prioritization of vaccination campaigns	more accurate predictive models for polio outbreaks
	faster response to outbreaks	
	strengthened routine immunization	
waning intestinal immunity?	studies to assess the importance of waning intestinal immunity for poliovirus persistence and potential strategies to boost mucosal immunity	improved understanding of the role of vaccinated children and adults in poliovirus transmission
vaccine-derived poliovirus (VDPV) outbreaks	coordinated OPV cessation	better understanding of risks of VDPV emergence and spread
	accelerated endgame strategy to sequentially remove poliovirus serotypes from OPV, starting with serotype 2	
	rapid response to VDPV outbreaks, equivalent to response to wild-type poliovirus	
re-emergence of poliovirus post- eradication	global action plan on poliovirus containment	safe and effective antiviral drugs
	screening of individuals with primary immunodeficiency for VDPV shedding	less transmissible (safer) seed strains for IPV manufacture
	recommended universal introduction of routine immunization with at least one dose of IPV	adjuvants or vaccine delivery technology to allow IPV dose reductions (reducing cost)
		immunogenicity of reduced dose IPV schedules
		non-transmissible vaccine that induces mucosal protection

## 2. Challenges to the eradication of wild-type poliovirus

### (a) Poor immunogenicity of OPV in certain populations

The live-attenuated OPV induces protective antibodies and immune memory by mimicking natural infection with poliovirus but with a significantly reduced probability of causing disease. Approximately one case of vaccine-associated paralytic poliomyelitis (VAPP) occurs per 750 000 doses of trivalent OPV for the first dose given [10], compared with one case of poliomyelitis per 100 to 1000 infections with wild-type poliovirus, depending on the serotype [11–13]. Early attempts by different institutes to develop genetically stable vaccine strains for each of the three poliovirus serotypes resulted in variable infectivity and virulence phenotypes [4]. Ultimately seed strains developed by Albert Sabin and his team were chosen for licensing on the basis of their infectivity and lower neurotropism in monkeys.

During the development of attenuated vaccine candidate strains it became apparent that immunogenicity varied according to the study population. During a mass vaccination campaign carried out in 1958 in what was the Belgian Congo with the serotype 1 CHAT strain (developed by Hilary Koprowski), it was noted that seroconversion was lower compared with that observed in large field studies with the same vaccine in Poland [14]. Poorer immunogenicity of OPV in lower-income settings has since been confirmed in numerous studies [15]. The resulting poor efficacy of OPV in these settings has been a major challenge to the global eradication of poliomyelitis. For example, until 2011 polio persisted in northern India despite frequent mass vaccination campaigns, where it acted as a reservoir of infection for the rest of the country. In the northern state of Uttar Pradesh, the efficacy of trivalent OPV against serotype 1 or 3 poliomyelitis was estimated using case–control methods at just 9 per cent per dose [16]. The significantly lower efficacy of trivalent OPV in Uttar Pradesh compared with other parts of India was a major reason for polio persistence at that time.

The GPEI responded to the challenge of poor immunogenicity of trivalent OPV in a number of ways. In 2005 serotype 1 and 3 monovalent OPVs were developed and licensed through a public–private partnership [17]. The trivalent formulation of OPV was known to result in interference between the vaccine strains, although the mechanisms underlying the observed interference have yet to be elucidated (competition for the poliovirus receptor and/or induction of innate antiviral immunity may play a role). Despite the development of a ‘balanced’ formulation of the trivalent vaccine [18], which contains less serotype 2 vaccine virus compared with the other serotypes, preferential seroconversion to this serotype is commonly observed. This effect is pronounced in lower-income settings, where the overall immunogenicity of OPV is diminished. In a review of 32 studies from lower-income countries Patriarca *et al*. [15] found that 27 per cent and 30 per cent of children lacked detectable serum neutralizing antibodies to serotype 1 and 3, respectively, after three doses of trivalent OPV. This compared with 10% for serotype 2 and explains why serotype 2 but not 1 and 3 could be eradicated with trivalent OPV. Sabin's serotype 2 vaccine strain appears to be a fitter virus, capable of out-competing the other two strains during replication in the human intestine. The immunogenicity of the serotype 1 and 3 vaccine strains can thus be increased simply by administration in the absence of serotype 2 (bivalent formulation) or on their own as monovalent vaccines.

OPV was initially developed and used as a series of monovalent vaccinations in the US during 1961–1963 and monovalent schedules continued to be used in some countries until the 1980s (e.g. East Germany, Hungary [19,20]). The reintroduction and licensing of monovalent vaccines in 2005, and in particular, the use of type 1 monovalent OPV to prioritize eradication of the most pathogenic serotype led to a significant increase in population immunity to that serotype in endemic countries and a reduction in serotype 1 poliomyelitis followed. The efficacy of this newly licensed vaccine in India was shown to be approximately three times greater per dose compared with trivalent OPV against serotype 1 [21], and this was subsequently confirmed in an immunogenicity study in Egypt [22] and further efficacy studies elsewhere [23,24].

Enthusiastic use of serotype 1 monovalent OPV during 2005–2009 led to a decline in poliomyelitis due to this serotype globally (figure 1). However, the overall impact on the global polio case count was limited because of a resurgence of serotype 3—a result of infrequent activities using vaccine containing this serotype (either monovalent or trivalent). The persistence of serotype 1 despite monovalent vaccine use, albeit at lower levels than before, motivated the introduction of a serotype 1 and 3 bivalent OPV in late 2009. The immunogenicity of this vaccine against both serotypes was shown to be only slightly less than the corresponding monovalent vaccines, indicating that serotype 2 Sabin virus is the major cause of interference in the trivalent formulation [25]. Bivalent OPV was first used in December 2009 in Afghanistan and was swiftly adopted more widely. In India it was first used in January 2010. A year later, elimination of remaining serotype 1 and 3 wild-type poliovirus was achieved and in 2012 India was declared ‘polio-free’. Critical to this success was the development and licensing of monovalent and bivalent OPVs [26].

Although more immunogenic compared with trivalent OPV, monovalent and bivalent formulations remain less immunogenic when administered to children in lower-income compared with higher-income countries [27]. Indeed, interference among vaccine strains only became a problem in those settings where the overall immunogenicity of the individual vaccine strains was compromised. This remains a challenge to the GPEI because it substantially increases the number of doses of OPV that need to be administered to achieve a high probability of protective immunity. Routine schedules where four or five doses of trivalent OPV are administered are not always sufficiently protective, and the impact of mass campaigns with OPV of any valency is blunted. It is striking that at the time of polio elimination in northern India, children under 5 years old had received on average 19 doses of OPV. Research into the causes of OPV failure and interventions to improve its immunogenicity is therefore ongoing.

### (b) Vaccination coverage

The original resolution made at the World Health Assembly to eradicate poliomyelitis was built on a vision of improving coverage of routine immunization and emphasized that ‘eradication efforts should be pursued in ways which strengthen the development of the Expanded Programme on Immunization as a whole’ [3]. However, in areas with weak health systems, mass vaccination campaigns or ‘supplementary immunization activities’ (SIA) were essential if coverage was to rapidly reach the level needed for global eradication by the year 2000. Besides, mass campaigns had already been used to successfully eliminate or control polio in several countries (e.g. Cuba, Brazil; [28,29]). Of course, in areas with weak health systems it can also be difficult to organize effective SIA. This challenge is most apparent in the three countries that remain endemic in 2013, where SIA either do not occur frequently enough, or occur with low coverage in the key reservoir areas of the virus.

Assessing coverage of SIA is itself a difficult task, but essential if programme performance is to be monitored and improved. A number of different approaches have been taken by the GPEI, including coverage estimates based on administrative records, vaccine stocks and distribution, independent monitoring of campaigns based on the presence of ink marked on the little finger at the time of vaccination, written and oral vaccination histories taken at the time of acute flaccid paralysis (AFP) case investigation and surveys based on different methodologies such as Lot Quality Assurance Sampling (LQAS). These different approaches to assessing coverage can result in highly discrepant estimates, with administrative, stock-based and independent monitoring all giving much higher estimates of coverage compared with LQAS or AFP data. The latter tend to give a clearer indication of programme performance and have been shown to correlate with the occurrence of polio cases [30].

The causes of poor vaccination coverage can be grouped into several key areas, although there is extensive interaction between them. In some countries there has been weak management and oversight of the programme at the local level, particularly in Nigeria and Pakistan [31]. This has limited the performance and monitoring of SIA. Poor management leads to vaccination teams that are incapable of delivering OPV to enough of the population. For example, teams in some parts of Pakistan have included only temporarily employed staff with insufficient local knowledge and no appropriate language skills, or they have lacked female members thereby limiting access to households when the men are absent [32]. In addition, SIA implementation and monitoring rely on accurate maps and census data (‘microplans’), which can be a major challenge, particularly for poorly managed programmes with insufficient technical support. Difficulties with programme management typically occur where political support is lacking, and this has been most notable at the provincial and district level in large federated republics such as Nigeria, India and Pakistan.

**Figure 2.**
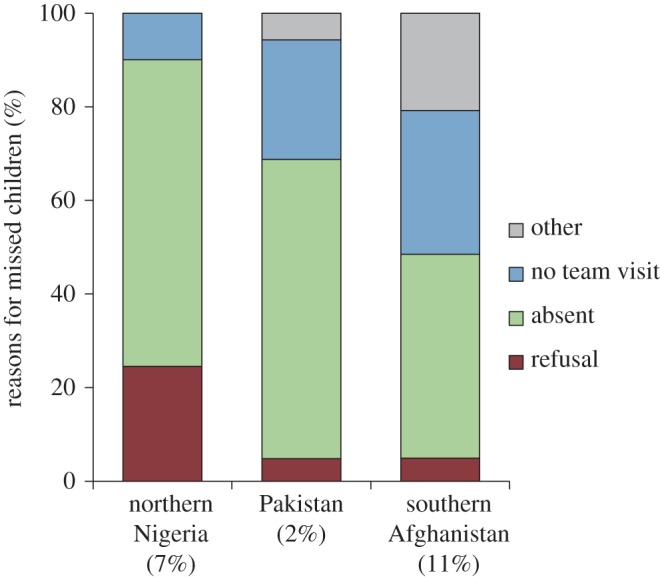
Reasons for missed children during SIA in the first half of 2012 based on independent monitoring data from the three regions that have yet to interrupt indigenous poliovirus transmission: southern Afghanistan, Pakistan and northern Nigeria. The proportion of all children 0–4-years old who were identified as missed is shown by the figures in brackets. In southern Afghanistan the ‘other’ category specifically refers to cases where the child was a neonate, asleep or sick. Southern Afghanistan includes Kandahar, Helmand, Urozgan, Zabul and Nimroz provinces. Northern Nigeria includes Bauchi, Borno, FCT Abuja, Gombe, Jigawa, Kaduna, Kano, Katsina, Kebbi, Plateau, Sokoto, Yobe and Zamfara states. Data courtesy UNICEF PolioInfo (www.polioinfo.org).

Resistance to immunization and lack of demand for OPV in some populations has also been a challenge, although resistance has not been as widespread is as sometimes suspected. The most notable and well-cited example is the polio vaccine boycott that occurred in some states of northern Nigeria in 2003, following rumours that the vaccine contained sterilizing compounds [33]. Generally, parents who refuse to vaccinate their children represent a small proportion (less than 10%) of the population. Independent monitors of SIA coverage routinely collect information on why children were missed by the campaign. Missed children are typically classified as refusals, absent at time of vaccination team visit, no team visit or ‘other’. Refusals tend to be one of the least common reasons for missed children (figure 2). However, a child who was missed because of ‘absence’ or ‘other’ reasons may often have been truly missed because of underlying resistance to immunization. Of course, the interpretation of why some parents refuse vaccination must be taken not just in the social and political context, but also in the context of the quality of the vaccination programme. A poorly managed programme with inappropriate vaccination teams can lead to refusals and ‘absent’ children.

Access to children can be a challenge among migratory populations, where there is active conflict or where populations are geographically isolated. For example, polio elimination in India was undermined not only by poor OPV immunogenicity, but also by difficulties in reaching children in inaccessible regions, such as the Kosi river flood plain, and in consistently vaccinating children living in migratory families.

Active conflict in the Federally Administered Tribal Areas (FATA) of Pakistan and in southern Afghanistan currently limits accessibility of children to vaccination teams. SIA are often cancelled or are simply not planned because of risks to programme staff, and as a result children may remain inaccessible for months at a time. More generally, insecurity and safety concerns limit the capacity of the GPEI to operate and put staff at risk. The deaths of programme staff in Pakistan and Afghanistan highlight the very real dangers of operating in these areas.

Finally, the GPEI has faced continuing ‘funding gaps’ that have resulted in scaling back of immunization activities and technical support to countries [31]. At the time of writing, the 2012–2013 Global Emergency Action Plan, budgeted at US$2.19 billion for core costs, planned SIA and emergency response, was facing a $790 million funding gap [34]. The narrower geographical extent and frequency of SIA that result from these financial constraints puts countries at risk of outbreaks as each year the number of susceptible, unvaccinated children increases. In the first half of 2012, a significant number of planned SIA in West Africa, Europe and South East Asia were cancelled because of a lack of funds [35].

The GPEI has responded to the challenges that limit vaccination coverage during SIA and continue to innovate in this area. Weak programme management and oversight was identified by the GPEI Independent Monitoring Board in 2011 as a major challenge, and gave a mandate for substantial changes to the GPEI [36]. Building on this momentum, in 2012 the World Health Assembly declared polio eradication a ‘programmatic emergency for global public health,’ and urged polio infected countries to declare polio transmission a ‘national public health emergency’ [37]. As a result, a new organizational structure for the GPEI was adopted and a Polio Oversight Board established. At the local level, accountability of programme staff was increased through a number of measures including the introduction of new staff contracts and performance reviews. At the same time, technical assistance was ramped up with the activation of emergency operation centres and procedures at CDC, UNICEF and WHO. Important lessons have also been learned from the successful programme in India, and staff from the Indian National Polio Surveillance Project are now providing technical assistance in Nigeria and other polio-affected regions.

The political will needed to support the GPEI over the last decade has been generated through dedicated advocacy work at all levels, particularly through the support of Rotary International and UNICEF. Rotary International has engaged heads of state and political bodies including the African Union, Organization of the Islamic Conference, the Commonwealth and G8 [31]. As a result, support for the GPEI at the highest political levels has been extraordinary. At the local level, religious and political leaders have been engaged by the GPEI and attitudes to vaccination have become positive where once they were negative. For example, in tribal areas of northern Pakistan previously resistant to vaccination, Islamic scholars and leaders have issued fatwas in favour of vaccination with OPV.

Advocacy at the local level occurs within a broader framework of mass communication and social mobilization led by UNICEF. These efforts have proved critical in recent years to the successes of the GPEI. It has been estimated that in endemic regions where communication has been included as a key component of immunization strengthening, vaccination coverage has increased by an absolute 12–20% compared with baseline [38].

Shortly after the year 2000 it was apparent that more had to be done to reach inaccessible children. House-to-house visits by vaccination teams in addition to fixed booth activities were systematically introduced to SIAs by the GPEI. At the same time, technical assistance from WHO was increased in countries and by 2009 over 3000 WHO-funded staff were working in 70 infected or high-risk countries [31]. In the last few years, vaccination of migratory populations involved in temporary or seasonal employment has been recognized as critical to success in the remaining infected areas. For example, children from migratory populations were disproportionately represented among reported cases of poliomyelitis in the last few years before elimination from India. Extensive efforts to map and vaccinate these populations are now in place in remaining endemic regions. For example, in India 162 000 migratory sites such as brick kilns and construction sites were mapped, and 4.2 million children under 5 years old identified at these sites. These efforts have been complemented by innovations in vaccine delivery, such as the continual presence of vaccination teams at transit points and the analysis of data from GPS devices fitted to cool boxes used during house-to-house visits.

Access to children in countries affected by conflict has been achieved through negotiated ceasefires and strengthened community involvement [39]. As a result, polio has been eliminated from many of these countries (e.g. Cambodia, Colombia, El Salvador, Sri Lanka, etc.). In the FATA of Pakistan and in southern Afghanistan, these approaches have been less successful. Instead, the shifting conflict and insecurity there is carefully mapped and the programme is swift to provide OPV and other health interventions when security risks are considered acceptable. A short-interval additional dose (‘SIAD’) strategy has been adopted in these areas, whereby a second dose of monovalent or bivalent OPV is given within two weeks to maximize OPV immunogenicity during any window of opportunity. However, access can be infrequent and conflict in these areas remains a major challenge to the GPEI.

Finally, efforts to improve SIA coverage have been linked to routine immunization strengthening. As a ‘vertical’ programme, the GPEI has been criticized for its failure to invest more in improving underlying immunization and health services. However, the strong community involvement in polio eradication and the mobilization of resources from governments offer many opportunities for strengthening routine immunization, which is one of the founding principles of the GPEI [40]. GPEI staff are regularly involved in activities unrelated to polio, such as supporting outbreak response activities for other infectious diseases and intensification of routine immunization during child health weeks. Formal commitments to routine immunization strengthening were made in the GPEI Strategic Plan 2010–2012, including the commitment to contribute at least 25 per cent of field staff time to this activity in African countries experiencing frequent importation and outbreaks of wild poliovirus [41].

### (c) Surveillance

Surveillance for poliomyelitis relies on the reporting of children under 14 years old with AFP through a network of health providers. These children undergo a clinical and epidemiological assessment, including the collection of two stool samples within 14 days of the onset of paralysis, which are tested for the presence of poliovirus. Most countries implementing AFP surveillance currently meet the WHO target of at least one case of AFP reported each year per 100 000 children under 15 years old, although there can be significant variability at the subnational level. Currently more than 100 000 children with AFP are investigated each year, giving polio eradication one of the most comprehensive and sensitive surveillance networks in global public health.

The scale of the AFP surveillance effort, despite the annual incidence of just a few hundred cases of poliomyelitis, is a reflection of one of the challenges facing polio eradication. The differential diagnosis for AFP includes many different causes, such as Guillain–Barré syndrome, infection with other neurotropic viruses, transverse myelitis and trauma [42]. There is no single clinical case definition that combines high sensitivity and specificity [43]. A virological case definition was therefore adopted in most countries by 2001 based on virus isolation from the two stool samples collected after case identification. The resulting annual workload of over 200 000 stool samples is processed by the 145 laboratories that make up the Global Polio Laboratory Network (GPLN).

The time taken to isolate poliovirus and to distinguish wild-type from vaccine-related polioviruses (intratypic differentiation) necessarily leads to delays in case confirmation and response activities. In 2006, the GPLN introduced new testing algorithms that reduced the time to culture and report poliovirus isolates from 28 to 14 days, and intratypic differentiation from 14 to 7 days [44]. These innovations in the laboratory have accelerated case confirmation and permitted more rapid outbreak response and mop-up activities. The performance standard for five of the six WHO regions is to report poliovirus isolation in at least 80 per cent of specimens within 14 days of receipt and to report intratypic differentiation results within 60 days of the onset of paralysis (allowing time for case detection, investigation and transport of specimens; [45]). This standard was met in each of these regions in 2011.

The sensitivity of AFP surveillance to detect poliovirus circulation is inherently limited because poliomyelitis only occurs in one case per 100 to 1000 infections [11–13]. In vaccinated populations the number of asymptomatic infections may be even higher as a result of asymptomatic infection and shedding of poliovirus among previously immunized individuals [46]. Poliovirus transmission can therefore occur in a population for several months or even a year or more without detection [47]. This contrasts strongly with smallpox, which is clinically apparent in almost every infection. As a result, if any ‘polio-free’ country detects a single child with poliomyelitis caused by wild-type poliovirus, it is considered a public health emergency and a large-scale outbreak response using monovalent OPV within four weeks and targeting 2–5 million children under 5 years old is recommended [48]. The ring vaccination strategy that was so successful in the final stages of smallpox eradication is not an option for the GPEI because poliovirus infection is typically far more widespread than the immediate social network of children with poliomyelitis.

Despite these limitations, AFP surveillance does permit high-risk areas for poliovirus transmission to be identified and targeted by SIA. Infected countries plan their SIA in advance (typically by six months) and AFP surveillance informs the number and location of SIA as well as the choice of vaccine type. The immunization histories of children with AFP as a result of causes other than infection with poliovirus (‘non-polio’ AFP) are also used by the GPEI to monitor vaccination coverage, estimate population immunity to each serotype and help predict trends in population immunity under different proposed SIA schedules [21,41].

The value of AFP surveillance is increased enormously by the routine sequencing of approximately 900 nucleotides in the VP1 region of all isolated wild-type and non-Sabin-like (vaccine-derived) polioviruses. Poliovirus is a single-stranded RNA virus that evolves at the very fast rate of approximately one substitution per 100 nucleotides per year [49]. Phylogenetic analysis of sequence information from children with poliomyelitis therefore allows inference of routes of spread of the virus at quite a fine spatial and temporal resolution [50]. Within countries these data may help identify migratory routes and socio-economic links among populations, which are important determinants of virus spread and persistence. Vaccination tactics can then be tailored to achieve high coverage among migratory populations.

**Figure 3.**
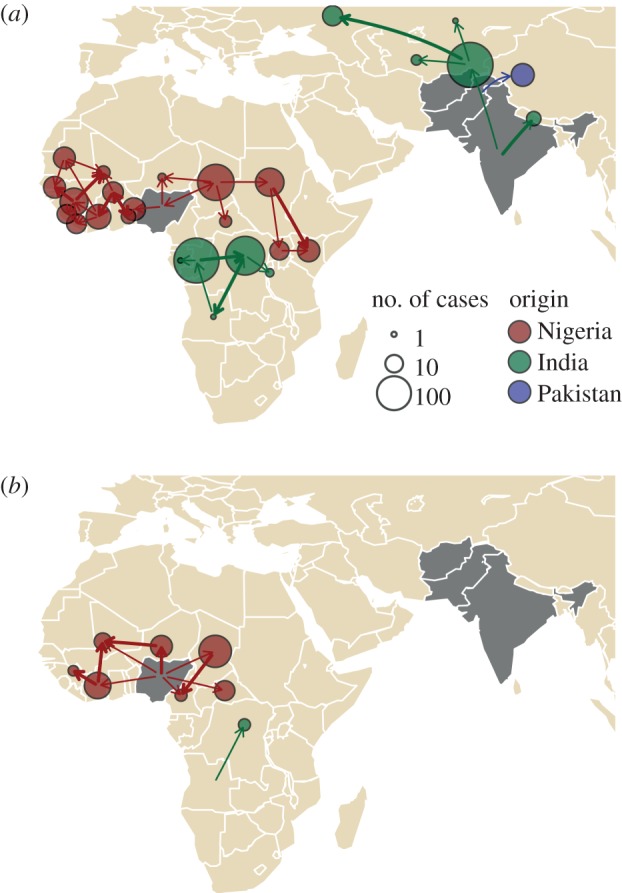
International spread of (*a*) serotype 1 and (*b*) serotype 3 wild polioviruses resulting in cases during 2009–2011 based on genetic sequencing information. The arrows indicate the direction of wild-type poliovirus spread and the circles are drawn in proportion to the number of cases that resulted from the importation of virus. Arrows and circles are colour-coded according to the original endemic country source of the virus. Endemic countries during 2009–2011 are shown in grey. Thicker arrows indicate more than one importation during the period of the analysis. At least 83 importations of wild-type poliovirus were detected during this period but many more such events would have occurred without detection of symptomatic cases. The origin and destination of the arrows point towards the centre of each country rather than the regions with circulation except in the case of China and Russia. Plot based on data presented in Kew *et al*. [135].

Genetic information also allows identification of novel polioviruses in a country and the likely origin of these viruses (figure 3). For example, during 2005–2007 wild poliovirus from northern India was repeatedly introduced to Angola, reflecting an economic tie associated with the oil industry [51]. The observed genetic diversity seen among wild-type polioviruses is informative about the extent of asymptomatic transmission and progress towards eradication can be tracked through assessment of trends in genetic diversity (usually by the number of genotypes or genetic clusters). Genetic sequence information can also be used to assess the sensitivity of AFP surveillance. Any wild-type poliovirus that is more than 1 or 2 per cent divergent in the VP1 region from the most closely related isolate is defined as an ‘orphan’ poliovirus and considered indicative of low AFP surveillance sensitivity. For example, during 2005–2007 genetic sequence analysis of wild-type polioviruses isolated from children with AFP in Pakistan and Afghanistan revealed 11 orphan viruses showing at least 2 per cent divergence from their most closely related isolates, indicating significant gaps in AFP surveillance [52].

Although reporting cases of AFP remains the standard for poliovirus surveillance, environmental surveillance is playing an increasingly important role. Depending on the setting, testing of sewage and wastewater samples for the presence of polioviruses can be far more sensitive than surveillance for cases of AFP in a community [53–56]. In areas with a convergent sewage network it has been estimated that a single 400 ml grab sample from the sewage system could detect poliovirus excretion by just 1 in 10 000 individuals [57]. Even in areas with rudimentary sewage systems, samples taken from canals that collect wastewater from the population of interest can offer a sensitive surveillance method. In urban India these methods detected wild-type polioviruses in the absence of AFP reports, and the absence of wild-type polioviruses in environmental samples offered the reassurance necessary to declare India ‘polio-free’ in early 2012 [58]. Genetic sequencing of polioviruses detected in sewage allows their probable origins and past history to be inferred through phylogenetic and population-genetic analysis. Together, environmental surveillance and genetic sequencing are therefore able to identify ‘silent’ circulation of wild-type polioviruses (in the absence of cases of poliomyelitis) and probable routes of spread. In countries currently free of wild-type polioviruses, environmental surveillance can provide an early warning of wild-type or vaccine-derived poliovirus infections in the population before any reporting of paralytic cases. In these countries isolation of vaccine-derived and wild-type polioviruses from environmental samples is quite frequently reported, both where OPV continues to be used (and rates of Sabin poliovirus isolation are high) [59–63] and in countries using only IPV [64,65]. However, these isolations have not so far been followed by outbreaks of poliomyelitis.

The GPEI plans to expand the number of environmental surveillance sites to improve poliovirus surveillance sensitivity, particularly in the post-eradication era when emergent vaccine-derived or wild-type polioviruses must be swiftly detected. However, environmental surveillance does face a number of limitations. Perhaps most importantly, sensitivity drops precipitously in areas that do not have convergent sewage networks. Rural or low density populations are therefore not amenable to environmental surveillance. In addition, current methods for processing sewage samples are laborious and the GPLN capacity to test environmental samples in addition to stool samples from children with AFP is limited. Several research groups are therefore pursuing more efficient sewage samplers and laboratory protocols to enhance the GPLN capacity in this regard.

### (d) Outbreaks

In the absence of wild poliovirus circulation and with the focus of most SIA on high-risk areas, the number of unvaccinated children susceptible to poliomyelitis has been increasing in many parts of the world—particularly in populations with limited access to routine immunization services. These populations are therefore at risk of poliomyelitis outbreaks. In Africa between July 2003 and the end of 2010, there were 137 outbreaks in 25 countries as a result of wild-type poliovirus importations detected by AFP surveillance [30]. Some of these outbreaks included a significant number of cases among older children and adults, reflecting the build-up of susceptibility in these populations [66].

The size distribution of outbreaks detected in Africa is highly skew, with most associated with only a single case of poliomyelitis but a small number associated with tens or hundreds of cases. The observed distribution is difficult to explain unless the majority of poliovirus importations (perhaps up to 90%) result in limited transmission and fade out before they are detected by AFP surveillance [67]. Indeed, environmental surveillance in many parts of the world confirms the frequent detection of wild-type polioviruses in the absence of AFP cases [45]. Populations with immunity gaps are therefore likely to be exposed to importations of wild-type poliovirus and are at risk of outbreaks of poliomyelitis.

The risk of poliomyelitis outbreaks can be forecast with reasonably high predictive ability using known risk factors including vaccination coverage and estimated exposure to imported polioviruses based on estimates of population movement from wild poliovirus-infected countries [30]. Preventive SIAs can therefore be planned to reduce emerging risks and this approach has been taken by the GPEI [41].

Population immunity gaps to poliovirus are reduced by secondary spread of vaccine poliovirus following administration of OPV [68]. Communities with low vaccination coverage may have a high prevalence of serum neutralizing antibodies as a result of secondary spread of OPV from neighbouring communities and contacts [69]. The extent of secondary OPV spread depends on factors affecting transmission of poliovirus such as sanitation and crowding. In some communities, secondary spread of OPV appears to be an important route towards providing population immunity. This is apparent in results from a clinical trial of monovalent and trivalent vaccines in India, where between 5 and 17 per cent of infants seroconverted to poliovirus serotypes other than those contained in the vaccine in the first 60 days of life [25]. Secondary spread of OPV can limit to some degree the build-up of susceptible children and adults in OPV-using countries, and is quite distinct from immunization programmes for other childhood infectious diseases such as measles.

In countries that have recently changed their routine immunization schedules to include IPV rather than OPV, secondary immunization of contacts no longer occurs. Communities with poor vaccination coverage may therefore be at increasing risk of a poliomyelitis outbreak following importation of virus. Long-term experience with IPV schedules mostly comes from high-income countries with good sanitation. Outbreaks of poliomyelitis in these countries have been restricted to small unvaccinated communities and limited in size [70–72]. However, middle-income countries with gaps in routine immunization coverage are now beginning to switch to IPV following long-term elimination of wild-type polioviruses. In these settings, the risk of large poliomyelitis outbreaks will be greater.

### (e) Waning intestinal immunity?

Intestinal immunity induced by oral poliovirus vaccine (OPV) is only partially protective against poliovirus replication and shedding in stool [73,74]. Children immunized with OPV may therefore be reinfected with poliovirus on subsequent exposure, while remaining immune to paralytic disease. A recent study of poliovirus shedding among OPV-vaccinated children in India following a subsequent ‘challenge’ dose of OPV found evidence for waning of intestinal immunity to poliovirus over a fairly rapid timescale [75]. Within a year, the odds of viral shedding following challenge increased by approximately 30–100% depending on serotype. These results suggest that older, OPV-vaccinated children may participate in the transmission of polioviruses as a result of incomplete, waning intestinal immunity. Consistent with this possibility, in India serotype 1 wild-type poliovirus was isolated from 15 per cent of healthy children after contact with children with poliomyelitis, despite previous vaccination with at least 10 doses of OPV [76]. Wild-type polioviruses have also been isolated in stool samples randomly sampled from older, school-aged children in Nigeria [77]. However, the quantity of wild-type poliovirus shed by these children is likely to be substantially less compared with unvaccinated children [78], and epidemiological data supporting participation of OPV-vaccinated children with waning intestinal immunity in wild-type poliovirus transmission are limited [46].

Adults may be reported with poliomyelitis during outbreaks in countries where vaccination coverage was historically low or interrupted by war or unrest (e.g. Congo; [66]). Adults may also contribute to transmission of wild-type polioviruses due to waning intestinal immunity. As for OPV-vaccinated children, however, the extent of this contribution is not clear, and behavioural factors may limit their infectiousness despite demonstrations of virus shedding in this age group. In Angola, a case-control study identified travel of an adult household member outside the province of residence as a risk factor for poliomyelitis, implicating adults in transmission of the virus [51]. Additional studies are needed in different settings to further assess the importance of adults for poliovirus transmission. If OPV-vaccinated adults and/or older children are found to play a significant role in wild-type poliovirus transmission, strategies to boost their intestinal immunity will be warranted. This could include the use of inactivated vaccine, which has been shown to boost mucosal immunity among individuals previously exposed to live polioviruses and does not suffer poor immunogenicity in lower-income settings [79].

## 3. Challenges to the eradication of all polioviruses

### (a) Vaccine-derived polioviruses and OPV cessation

The Sabin poliovirus strains in use today were attenuated through an empirical process involving serial passage in cell culture and primates and assessment of neuropathogenicity in monkeys [4]. The molecular basis of attenuation was only elucidated more recently. A series of studies revealed just a small number of mutations were responsible for the reduced neuropathogenicity of each of the Sabin strains (reviewed in [80]). Furthermore, following administration of OPV, Sabin poliovirus rapidly evolves through a process of mutation and intertypic recombination to lose these attenuating mutations [81,82]. Loss of attenuating mutations is frequently found among vaccine polioviruses isolated from children with VAPP, following administration of OPV [83,84]. The problem of VAPP was recognized within a year of licensure of OPV [85]. It also became apparent that VAPP could occur among contacts of individuals who had received OPV as a result of secondary spread of the vaccine poliovirus. Indeed, the number of children with suspected ‘contact’ VAPP is typically equivalent to or can exceed the number of cases of ‘recipient’ VAPP [86,87].

The theoretical possibility of outbreaks of poliomyelitis as a result of sustained transmission of vaccine-derived polioviruses has long been recognized [2]. This risk remained theoretical until the year 2000, when genetic sequence analysis of poliovirus isolated during an outbreak of poliomyelitis in Haiti and the Dominican Republic found a serotype 1 Sabin origin for the virus [7]. Recognition of this outbreak of vaccine-derived poliovirus (termed VDPV) was followed by further outbreaks of independently evolved VDPVs in other countries and by retrospective identification of VDPVs from stored stool samples associated with past outbreaks of poliomyelitis [88,89]. Improved laboratory methods based on real-time PCR with primers specific to nucleotide substitutions typically observed early in VDPV evolution are now used widely within the GPLN to identify emergent and circulating VDPV from stool and environmental samples [90]. The GPEI labels a vaccine-derived poliovirus as ‘circulating’ (a cVDPV) when it is at least 1 per cent divergent from Sabin in the VP1 region (0.6% for serotype 2) and where at least two children with AFP shed closely related virus. At the time of writing 20 cVDPV had been reported from 20 different countries, the vast majority derived from the serotype 2 Sabin strain [91] and many recombinants with other human enteroviruses [92,93]. The predominance of serotype 2 VDPV is the result of the greater fitness of this virus compared with the other Sabin strains and declines in population immunity to serotype 2 following the introduction of monovalent and bivalent OPVs.

In a study of the largest outbreak recorded to date, serotype 2 VDPV in Nigeria was found to have a similar attack rate to co-circulating wild-type polioviruses [8]. Similar results were found for an outbreak of a serotype 1 VDPV in Indonesia [94]. It is therefore clear that VDPVs can evolve to regain equivalent transmissibility and pathogenicity to wild-type polioviruses, and outbreaks must be responded to with the same vigour.

The identification of poliomyelitis outbreaks caused by VDPVs made apparent the risks of continued OPV use post-eradication of wild-type polioviruses. At a consultation held by the WHO in 2003, it was agreed that OPV use would have to cease post-eradication and that a clear ‘endgame’ strategy was needed [95]. Coordinated global cessation of OPV, 3 years after the last wild-virus-associated case of poliomyelitis was identified as a strategy that could minimize risks by stopping the introduction of vaccine poliovirus into the environment at a time when population immunity and the sensitivity of AFP surveillance are at their maximum [96]. Experience from Cuba and Mexico had shown that after mass immunization with trivalent OPV, and in the absence of routine vaccination with OPV, Sabin polioviruses disappeared from stool and environmental samples within 4–5 months [97–99]. More recently, countries switching to routine immunization with IPV have also documented disappearance of Sabin polioviruses several months after national immunization days or cessation of OPV [100,101].

In 2011, a revised endgame strategy was proposed by the GPEI, allowing for phased removal of Sabin poliovirus serotypes [102]. Over the last decade just over 500 children had been reported with poliomyelitis as a result of serotype 2 cVDPVs (approximately 90% of all documented poliomyelitis cases due to cVDPVs) and more than 1500 children were estimated to have developed VAPP as a result of the serotype 2 component of the trivalent vaccine [91,103]. Yet circulation of serotype 2 wild-type poliovirus was last recorded in 1999. Removal of serotype 2 Sabin poliovirus from all routine and SIAs would prevent these cases of poliomyelitis while providing an opportunity to eradicate one of the three poliovirus serotypes at a time of heightened surveillance and outbreak response capacity. This accelerated endgame strategy could begin as soon as currently circulating serotype 2 VDPVs are eliminated, raising the possibility of global eradication of at least one poliovirus serotype in the near future.

### (b) Re-emergence

Following global cessation of vaccination with OPV, risks of poliovirus re-emergence come from a number of sources. In the first 2 or 3 years after cessation, there is a risk that vaccine-derived polioviruses derived from OPV given prior to cessation could circulate at low levels before re-emergence with a revertant wild-type phenotype. Similarly, there is a risk that wild-type polioviruses could circulate for several years without detection of AFP [47]. However, the magnitude of these risks is probably quite small and is likely to diminish quite rapidly with time [104].

Other risks will persist in the longer term. Children and occasionally adults with primary B-cell immunodeficiencies can shed VDPV for several years following administration of OPV (so called iVDPV) [89]. Although OPV is not indicated for children with primary immunodeficiencies (cf. acquired immunodeficiency syndrome), it may be administered before the condition is diagnosed or result from secondary spread of the vaccine virus [105]. Individuals shedding iVDPV could in theory initiate an outbreak of VDPV—a risk supported by the identification of vaccine-polioviruses in environmental samples with genotypes characteristic of iVDPV [65,106]. However, no poliomyelitis outbreaks to date have been attributed to an iVDPV origin. Furthermore, the number of immunodeficient patients shedding iVDPV identified by the GPEI is relatively small (approx. 65 had been identified by June 2012) and largely restricted to higher- and upper-middle-income countries because of the complex medical needs of these patients [90]. Indeed, the majority of individuals shedding iVDPV are only identified as a result of VAPP and many have since died from poliomyelitis or other causes.

The GPEI is working with lower- and middle-income countries to screen individuals with primary immunodeficiency for poliovirus infection to quantify and mitigate the risks of iVDPV spread [107–109]. Extensive efforts are also underway to develop antiviral drugs that can be used to provide treatment and prevent shedding of iVDPV in these cases [110].

Persistent risks of poliovirus re-emergence are also presented by laboratory stocks that may contain wild or vaccine-related polioviruses [111]. To minimize these risks the GPEI will coordinate destruction of all materials that contain or potentially contain infectious poliovirus in non-essential facilities, and enforce strict biosafety measures at designated essential facilities such as diagnostic laboratories. National inventories of facilities with infectious or potentially infectious materials have already begun as part of the detailed global action plan on poliovirus containment [112].

Manufacture of IPV presents a risk of re-introduction of polioviruses, since most manufacturers currently use wild-type poliovirus seed strains. To reduce this risk an extensive research programme to develop alternative seed strains for IPV has been supported by the GPEI. IPV manufactured from Sabin seed strains has been shown to be immunogenic [113] and presents a lower risk of poliovirus transmission following any accidental release because of the significantly higher median infectious dose for Sabin compared with wild-type polioviruses [111]. Further attenuated strains are also under development as seed strains, based on changes to the 5′ non-coding region [114] or ‘codon de-optimization’ where multiple, unpreferred synonymous mutations are introduced to the capsid region [115,116].

Theoretically, there is also a risk of deliberate release of poliovirus, although this risk is difficult to assess. Even if poliovirus stocks are ultimately destroyed, the simplicity of the poliovirus genome makes it simple to synthesize in the laboratory using non-natural templates [117]. Similarly, although there is no non-human reservoir for poliovirus, in populations devoid of poliovirus antibodies a poliovirus could evolve again from its C cluster coxsackie A virus (CCAV) ancestors through mutations in the capsid region, which determines receptor specificity [118]. However, the number of amino acid substitutions separating the CCAV capsid, which binds to intercellular adhesion molecule-1 (ICAM-1), and the poliovirus capsid, which binds to the poliovirus receptor (CD155), is large and the replicative fitness of intermediates and likelihood of re-emergence through mutation unclear.

The current strategy for outbreak response after the eradication of wild-type polioviruses and OPV cessation is to implement mass vaccination campaigns with monovalent OPV corresponding to the serotype of any re-emergent poliovirus [96]. This strategy relies on the maintenance of a sensitive AFP surveillance network and an international stockpile of monovalent OPVs of sufficient scale to rapidly induce immunity in the population at risk. Its effectiveness will depend on where wild or vaccine-derived polioviruses re-emerge [119]. Re-emergence in a large, mobile population in an area with poor sanitation will present a major challenge, analogous to the final stages of wild virus eradication.

IPV has an obvious role in the protection of individuals against poliomyelitis in a post-OPV world. If introduced at the time of serotype 2 OPV withdrawal, or following cessation of all routine OPV use, routine immunization with IPV would protect children should poliovirus be re-introduced to the population. IPV is also likely to limit transmission of the re-introduced poliovirus, although its impact in lower-income settings with efficient faecal–oral transmission is less clear, given the more limited impact on poliovirus shedding in stool compared with OPV [120]. Introduction of IPV (together with bivalent OPV) to routine immunization programmes at the time of serotype 2 OPV withdrawal could also boost immunity to serotypes 1 and 3 [121,122]. This could help eradicate wild-type polioviruses, although only in areas where routine immunization coverage is high [123].

The major challenge to widespread introduction of IPV to routine immunization schedules at the time of OPV cessation has been its cost. For this reason, universal adoption of IPV following OPV cessation has not previously been recommended by WHO [124]. However, a number of initiatives to rapidly develop an affordable IPV for lower-income countries are currently underway, supported by the GPEI. These are mainly focused on dose-sparing strategies by intradermal administration [125–128] and/or use of adjuvants [129,130]. These strategies may be combined with a reduced schedule of just one or two doses, which would prime the majority of children, even in the absence of seroconversion (depending on the age at administration; [131]). Primed children are likely to be protected against poliomyelitis, although there is limited and somewhat conflicting evidence as to the degree of protection. Although a number of regulatory hurdles remain for the licensure and use of these new vaccines, an affordable IPV option is considered feasible in the timeframe of the polio endgame [132]. In November 2012, the WHO Strategic Advisory Group of Experts on Immunization therefore recommended that all countries should introduce at least one dose of IPV in their routine immunization programmes to mitigate the risks and consequences associated with the eventual withdrawal of serotype 2 OPV [133]. In the longer term, combination vaccines containing IPV, whole cell pertussis and other antigens are likely to be a sustainable option and are currently under development by a number of manufacturers [134].

## 4. Conclusion

The global eradication of serotype 2 wild poliovirus demonstrates the feasibility of eradicating all wild-type polioviruses. Success against serotype 2 was achieved as a result of the greater immunogenicity of trivalent OPV against this serotype, particularly in lower-income countries [15]. There is no evidence that wild poliovirus serotypes 1 and 3 are more transmissible than serotype 2, indeed the opposite may be the case for serotype 3 [2]. The introduction of new monovalent and bivalent OPVs in 2005 and 2009, respectively, with immunogenicity equivalent to or exceeding that of the trivalent vaccine against serotype 2 therefore suggests that these serotypes can also be eradicated in the near future.

The global eradication of serotype 2 wild poliovirus also highlights some of the challenges that will be faced after the eradication of all wild-type polioviruses. In particular, with the increasing use of monovalent and bivalent OPVs against serotypes 1 and 3, gaps in population immunity to serotype 2 have led to increasing incidence of poliomyelitis caused by circulating serotype 2 VDPVs. These cVDPV result from the continued use of trivalent OPV during routine immunization and in limited numbers of SIA. The polio endgame strategy addresses this challenge by calling for global, coordinated withdrawal of OPV serotypes, and eventually of all OPV. The GPEI must eradicate all polioviruses, not just wild-type poliovirus. A clear strategy for the management of post-OPV risks is also being put in place, including continued AFP surveillance, the maintenance of an international monovalent OPV stockpile and policy guidance on routine immunization with IPV to mitigate risks following a poliovirus re-emergence.

The successful reduction of the global incidence of poliomyelitis from over 1000 cases a day in 1988 to less than one a day in 2012 is a major achievement of the GPEI. The endgame strategy is designed to take the world from low incidence to no incidence. There is every reason to believe that this is possible with the continued commitment of the global health community.
